# Berbamine Suppresses the Progression of Bladder Cancer by Modulating the ROS/NF-*κ*B Axis

**DOI:** 10.1155/2021/8851763

**Published:** 2021-01-13

**Authors:** Chenglin Han, Zilong Wang, Shuxiao Chen, Lin Li, Yingkun Xu, Weiting Kang, Chunxiao Wei, Hongbin Ma, Muwen Wang, Xunbo Jin

**Affiliations:** ^1^Department of Urology, Shandong Provincial Hospital, Cheeloo College of Medicine, Shandong University, Jinan, Shandong 250021, China; ^2^Department of Vascular Surgery, Shandong Provincial Hospital, Cheeloo College of Medicine, Shandong University, Jinan, Shandong 250021, China; ^3^Department of Orthopedics, Shandong Provincial Hospital, Cheeloo College of Medicine, Shandong University, Jinan, Shandong 250021, China; ^4^Department of Urology, Shandong Provincial Hospital Affiliated to Shandong First Medical University, Jinan, Shandong 250021, China; ^5^Department of Hepatobiliary, The First Affiliated Hospital of Harbin Medical University, Harbin, Heilongjiang 150000, China

## Abstract

Berbamine (BBM), one of the bioactive ingredients extracted from *Berberis* plants, has attracted intensive attention because of its significant antitumor activity against various malignancies. However, the exact role and potential molecular mechanism of berbamine in bladder cancer (BCa) remain unclear. In the present study, our results showed that berbamine inhibited cell viability, colony formation, and proliferation. Additionally, berbamine induced cell cycle arrest at S phase by a synergistic mechanism involving stimulation of P21 and P27 protein expression as well as downregulation of CyclinD, CyclinA2, and CDK2 protein expression. In addition to suppressing epithelial-mesenchymal transition (EMT), berbamine rearranged the cytoskeleton to inhibit cell metastasis. Mechanistically, the expression of P65, P-P65, and P-I*κ*B*α* was decreased upon berbamine treatment, yet P65 overexpression abrogated the effects of berbamine on the proliferative and metastatic potential of BCa cells, which indicated that berbamine attenuated the malignant biological activities of BCa cells by inhibiting the NF-*κ*B pathway. More importantly, berbamine increased the intracellular reactive oxygen species (ROS) level through the downregulation of antioxidative genes such as Nrf2, HO-1, SOD2, and GPX-1. Following ROS accumulation, the intrinsic apoptotic pathway was triggered by an increase in the ratio of Bax/Bcl-2. Furthermore, berbamine-mediated ROS accumulation negatively regulated the NF-*κ*B pathway to a certain degree. Consistent with our in vitro results, berbamine successfully inhibited tumor growth and blocked the NF-*κ*B pathway in our xenograft model. To summarize, our data demonstrated that berbamine exerts antitumor effects via the ROS/NF-*κ*B signaling axis in bladder cancer, which provides a basis for further comprehensive study and presents a potential candidate for clinical treatment strategies against bladder cancer.

## 1. Introduction

Bladder cancer is the 7th most common malignancy in males and remains the leading cause of urinary disease-related death [1]. An estimated 549,000 new cases diagnosed as bladder cancer and 200,000 deaths occurred worldwide in 2018 [2]. In terms of clinical and pathological aspects, urothelial carcinoma, which accounts for 90% of primary bladder malignant tumors, is the major histological subtype. Among those bladder cancer patients, non-muscle-invasive bladder cancer (NMIBC) accounts for 70% of diagnoses. Moreover, as many as 40% of NMIBCs eventually develop into muscle-invasive bladder cancer (MIBC), which is extremely aggressive and has overall 5-year and 10-year survival rates of 50% and 36%, respectively [3, 4]. To date, therapeutics such as surgical resection and chemotherapy, mainly based on the tumor's clinical stage, have made certain progress. However, some hurdles, including adverse side effects, drug resistance, and the high recurrence rate, restrict sustainable clinical benefits. Therefore, it is highly necessary to screen more effective alternatives with low toxicity and determine their underlying mechanisms for patients with BCa.

Chinese traditional herbs have a wide range of pharmacological effects for clinical applications, such as anti-inflammatory, lipid modulation, antivirus, and antitumor [5, 6]. Berbamine, initially identified as an effective antileukemic agent extracted from the herbal medicine *Berberis*, garnered much attention [7]. A large number of research achievements further corroborate the antitumor properties of berbamine and its derivatives in various carcinomas, such as colon cancer, ovarian cancer, prostate cancer, and liver cancer [8–11]. Nonetheless, there have been no reports so far involving the effects of berbamine on the biological activities of bladder cancer.

ROS are a class of highly reactive, oxygen-containing molecules, mainly including superoxide anion, hydrogen peroxide, hydroxyl radicals, and singlet oxygen [12, 13]. Regulation of the ROS signaling network is a complex process. Under physiological conditions, a moderate level of ROS guaranteed by redox balance is crucial to a series of biological processes. However, compared with normal cells, cancer cells inherently exhibit aberrantly higher ROS levels due to their high metabolic rate, which profoundly facilitates the onset and deterioration of various human cancers by mediating oxidative damage to DNA, proteins, and lipids. Due to their dualistic nature, ROS can exert opposite biological effects. Once the extremely high ROS level exceeds intracellular tolerance, it can induce mitochondrial dysfunction and destroy cellular homeostasis, ultimately eliciting apoptosis, which provides possible insights into cancer treatment [14].

The process of tumor progression is synergistic, involving various intracellular proteins and complex signal transduction. Emerging evidence has demonstrated that abnormal upregulation of the inducible transcription factor NF-*κ*B is closely associated with unfavorable prognosis in patients with MIBC [15–17]. In general, inactive P65 and P50 heterodimers were bound to the inhibitor I*κ*B*α* in the cytoplasm. For NF-*κ*B to be activated, I*κ*B*α* must undergo phosphorylation, ubiquitination, and degradation; then, free P65 is phosphorylated for nuclear translocation and binds to DNA sequences at the promoter region of downstream target genes to regulate cellular processes [18]. In cancer cells, NF-*κ*B activation initiates the transcription of proliferative, metastatic, and angiogenic genes, all of which contribute to carcinogenesis [19]. Therefore, targeting the NF-*κ*B pathway has emerged as an effective strategy for cancer therapeutics.

We aimed to investigate the broad-spectrum effects of berbamine on bladder cancer in vitro and in vivo and elucidate its underlying mechanism. Cell phenotype experiments have revealed that berbamine could inhibit bladder cancer cell survival, proliferation, and metastasis by suppressing the NF-*κ*B pathway. Moreover, berbamine could induce cell cycle arrest at S phase accompanied by alteration of P21, P27, CyclinD, CyclinA2, and CDK2 proteins. We further established that berbamine downregulated the expression of several key antioxidative genes and subsequently elicited mitochondrial ROS generation that ultimately mediated cell apoptosis and negatively regulated the NF-*κ*B pathway to a certain degree. Collectively, these findings indicated that berbamine could attenuate the multiple biological properties of bladder cancer by modulating the ROS/NF-*κ*B axis. This study improves our understanding of the antitumor mechanism of berbamine against bladder cancer, thereby providing a basis for further comprehensive studies.

## 2. Materials and Methods

### 2.1. Cell Lines and Culture Conditions

Bladder cancer cell lines (5637 and T24 cells) were purchased from the Chinese Academy of Sciences (Shanghai, China). The above cells were cultured at 37°C in a humidified incubator containing 5% CO_2_ in RPMI-1640 medium (Gibco, China) supplemented with 10% fetal bovine serum (Gibco, USA) and 1% penicillin (Sigma-Aldrich, Italy) and streptomycin (Sigma-Aldrich, Italy).

### 2.2. Cell Counting Kit-8 Assay (CCK-8)

The CCK-8 assay was applied to evaluate the viability of bladder cancer cell lines (Dojindo, Japan). Appropriate 5637 and T24 cells were cultured in 96-well plates overnight and treated at the indicated doses. Following a certain period, the supernatant solution was replaced by 110 *μ*l fresh medium containing 10 *μ*l CCK-8 solution; then, the cells were incubated for 2 h at 37°C. The absorbance of each well was measured with a microplate reader at a wavelength of 450 nm.

### 2.3. Colony Formation Assay

5637 and T24 cells were uniformly dispersed in 6-well culture plates at an approximate density of 1000/well. Cells were cultured with the indicated berbamine for 48 h, and the medium was renewed every three days. After a two-week cultivation, the colonies were fixed in 4% paraformaldehyde, stained with hematoxylin (Solarbio), and counted using ImageJ.

### 2.4. Wound Healing Assay

Exponentially growing cells were seeded in 6-well culture plates and allowed to reach approximately 95% confluence in complete medium. A sterile pipette tip was applied to scratch the cell layer to create a wound. Subsequently, the cells were cultured in serum-free medium containing a specified berbamine concentration. The images of wound closure were captured using an inverted microscope, and the healing rate was assessed by Image J.

### 2.5. Transwell Assay

Appropriate 5637 and T24 cells resuspended in serum-free medium (200 *μ*l) with a specific concentration of berbamine were placed in the Transwell chamber (24-well, 8 *μ*m pore membrane, Corning Incorporated, NY, USA). For cell invasion, the upper chamber membrane was precoated with Matrigel (Corning Incorporated, NY), not for cell migration. Subsequently, 500 *μ*l of medium containing 20% FBS was presented in the lower chamber. After incubation at 37°C for 48 h, the cells on the upper surface of the membrane were wiped with a cotton swab, and then migrated or invaded cells were fixed in 4% paraformaldehyde and stained with hematoxylin (Solarbio, China). The images were taken in five randomly selected fields by a microscope (Leica Microsystems, GmbH).

### 2.6. Cell Cycle Analysis

5637 and T24 cells were cultured for starvation overnight and then pretreated with berbamine for 48 h. Subsequently, the collected cells were resuspended and fixed with precooled 75% ethanol at 4°C overnight. Following incubation with 1 mg/ml RNase A at 37°C for 30 min, the cells were stained with the propidium iodide (PI) solution for 20 minutes in the dark. Ultimately, the distribution of the cell cycle phase was analyzed through flow cytometry using a BD FACSArray (BD Biosciences, USA).

### 2.7. Cell Apoptosis Analysis

The collected cells were resuspended in 100 *μ*l 1x binding buffer supplemented with 5 *μ*l Annexin V-FITC and 5 *μ*l propionate (BD.559763), followed by incubation at room temperature in the dark for 15 minutes. After staining, the cell apoptosis rate was calculated using flow cytometry (BD Biosciences, USA) and FlowJo 7.6.2 software. At least 10000 cells were guaranteed before analysis.

### 2.8. 5-Ethynyl-2′-Deoxyuridine (EdU) Assay

5637 and T24 cells were seeded in 24-well plates with berbamine treatment for 48 h and incubated with medium supplemented with 50 *μ*M EdU. Two hours later, the cells were fixed with 4% paraformaldehyde at room temperature for 20 minutes, permeabilized in 0.5% Triton X-100 for 10 minutes, and stained with Apollo staining solution and Hoechst reagent. Finally, images were taken using fluorescence microscopy (Olympus, Tokyo, Japan).

### 2.9. Phalloidin Staining

After berbamine treatment for 48 h, 5637 and T24 cells were fixed with cooled carbinol and incubated with 50 *μ*g/ml FITC-phalloidin (Sigma-Aldrich) at room temperature in the dark for 1 h. Next, the cells were counterstained with DAPI (Sigma-Aldrich, USA). Finally, cell morphology was observed under a fluorescence microscope (Olympus, Japan).

### 2.10. Cell Transfection

pcDNA3.1-P65 and the empty vector were obtained from Genomeditech (Shanghai, China). When the cell confluence was approximately 50% in 6-well plates, the OV-P65 plasmid and the empty vector were transfected into 5637 and T24 cells with Lipofectamine 3000 Reagent (Invitrogen, USA). After 48 h of transfection, the cells were collected for subsequent experiments.

### 2.11. Mitochondrial ROS Measurement

Briefly, berbamine-treated cells were incubated with 5 *μ*M MitoSOX reagent working solution at room temperature in the dark for 10 minutes. Then, the cells were fixed in 4% paraformaldehyde and stained with DAPI. Finally, images were captured using a fluorescence microscope (Olympus, Japan).

### 2.12. Immunofluorescence

Cells were fixed with 4% paraformaldehyde for 20 minutes and permeabilized in 0.5% Triton X-100 for 10 minutes. Next, the cells were blocked with normal goat serum for 1 h and incubated overnight with the primary antibody at 4°C, followed by incubation with the Alexa Fluor 488-conjugated secondary antibody in the dark for 2 h. Finally, the cell nuclei were counterstained with DAPI, and images were captured using a fluorescence microscope (Olympus, Japan).

### 2.13. Quantitative Real-Time PCR (qRT-PCR)

The total RNA was extracted from 5637 and T24 cells by using the TRIzol Reagent (Takara, China) and was subsequently reverse-transcribed into cDNA with the PrimeScript™ RT Reagent Kit (Takara) according to the manufacturer's instructions. qRT-PCR analysis was carried out using the TB Green™ Premix Ex Taq™ II (Takara). The primer sequences were as follows: GAPDH (forward: 5′-GCACCGTCAAGGCTGAGAAC-3′; reverse: 5′-TGGTGAAGACGCCAGTGGA-3′) and P65 (forward: 5′-GACGCATTGCTGTGCCTTC-3′; reverse: 5′-TTGATGGTGCTCAGGGATGAC-3′). The GAPDH gene was regarded as an internal reference for P65 mRNA. The relative expression levels were calculated by the 2^−ΔΔCt^ method. All trials were conducted in triplicate (3 wells).

### 2.14. Western Blotting

Total protein was extracted from bladder cancer cells using RIPA lysis buffer (CST, USA) with 1% phosphatase inhibitors and 1% protease inhibitors on ice, and then quantified with the bicinchoninic acid (BCA) method (Solarbio). Each sample (25 *μ*g) was separated by 10% SDS-PAGE, transferred to a polyvinylidene fluoride (PVDF) membrane, blocked with 5% skim milk powder, and incubated with the primary antibody overnight at 4°C. The next day, the membrane was incubated with the HRP-conjugated secondary antibody for 1 h and finally visualized using an enhanced chemiluminescence kit.

### 2.15. Xenografts

The animal protocol was approved by the Institutional Animal Care and Use Committee of Shandong University. A suspension containing 5 × 10^6^ T24 cells was injected subcutaneously into the right axilla of nude mice (specific-pathogen-free (SPF) grade, 4 weeks old) that were randomly divided into the control group and BBM group (*n* = 5 for each group). When the tumor volume in each nude mouse was greater than 100 mm^3^, the mice in the treatment group were intraperitoneally injected with berbamine at 35 mg/kg body weight every three days until the completion of the experiment. Simultaneously, the mice in the control group were exposed to the same concentration of DMSO. At the termination of the experiment, the mice were sacrificed by cervical dislocation, and solid tumors were removed for evaluation. In addition, a portion of tumor tissues were embedded in paraffin for immunohistochemistry (IHC).

### 2.16. IHC

Tumor tissues were fixed with 4% paraformaldehyde and embedded in paraffin for slicing. Subsequently, the samples were deparaffinized, rehydrated, and washed with PBS. These samples were immersed in the antigen retrieval solutions with 10 nM citrate buffer (pH 6.0) for 3 minutes and incubated with the Ki-67 antibody and P65 antibody at 4°C overnight. The next day, the sections were incubated with the biotin-conjugated secondary antibody for 1 h. According to the manufacturer's procedures, protein staining was carried out with the DAB enzyme (Abcam, ab64238), and the nuclei were stained with hematoxylin (Abcam, ab143166). The stained slides were observed under a microscope.

### 2.17. Statistical Analysis

All values are expressed as the mean ± SD. Prism software (GraphPad, USA) was used to do statistical analysis. Statistical significance was determined using two-tailed Student's *t*-test or one-way ANOVA. Differences with *p* values less than 0.05 were considered statistically significant.

## 3. Results

### 3.1. Berbamine Suppressed the Growth of Bladder Cancer Cells In Vitro

**Figure 1:**
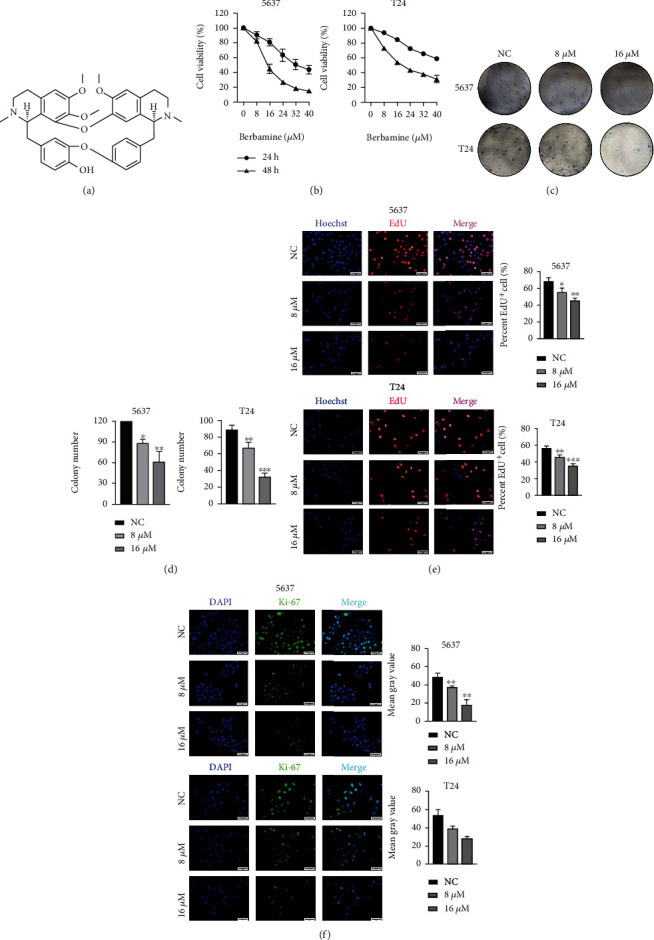
Inhibitory effects of berbamine on the proliferation of bladder cancer cells in vitro. (a) The chemical structure of berbamine was depicted. (b) A CCK-8 assay was conducted to evaluate the viability of 5637 and T24 cells treated with different concentrations of berbamine. (c, d) Representative images of colony formation assays and quantitative analysis of the numbers of colonies. (e) EdU assay: cell nuclear dye Hoechst (blue) and red fluorescence stands for DNA synthesis; percentage of EdU-positive cells of each group was calculated using a fluorescence microscope. (f) Representative images of the Ki-67 level in 5637 and T24 cells treated with berbamine. Green indicates Ki-67 intensity, and DAPI staining is for nuclei visualization. Values are represented (all dates are expressed) as the mean ± SD. The experiment was repeated at least three times. Statistical significance was determined using two-tailed Student's*t*-test or one-way ANOVA. ^∗^*p* < 0.5; ^∗∗^*p* < 0.01; ^∗∗∗^*p* < 0.001.

The chemical structure of berbamine is displayed in Figure 1(a). The CCK-8 assay was first performed to delve into the cytotoxic effects of berbamine. Briefly, cells were treated with a range of concentrations of berbamine (8, 16, 24, 32, and 40 *μ*M) for 24 h or 48 h, and cell survival was calculated in comparison with that of untreated cells. According to Figure 1(b), berbamine significantly suppressed the viability of both 5637 and T24 cells in a concentration- and time-dependent manner. The 50% inhibitory concentration (IC50) values of berbamine for 5637 and T24 cells at 48 h were 15.58 ± 2.489 and 19.09 ± 0.68 *μ*mol/l, respectively. Therefore, we applied suitable concentrations (8 *μ*M and 16 *μ*M) of berbamine to subsequent experiments. As shown in Figures 1(c) and 1(d), berbamine treatment significantly decreased the number of colonies compared to that in the control group. Additionally, the EdU assay visually suggested an antiproliferative activity of berbamine, as it disturbed DNA replication. Following berbamine treatment, the percentages of EdU-positive 5637 and T24 cells were markedly reduced (Figure 1(e)). Consistent with the EdU assay, immunofluorescence assays indicated that the level of Ki-67, a vital marker of cell proliferation, was notably decreased in both cell lines in response to berbamine (Figure 1(f)). In conclusion, the above outcomes illustrated that berbamine strongly restrained bladder cancer cell growth in vitro.

### 3.2. Berbamine Induced Cell Cycle Arrest at S Phase in Bladder Cancer Cells

**Figure 2:**
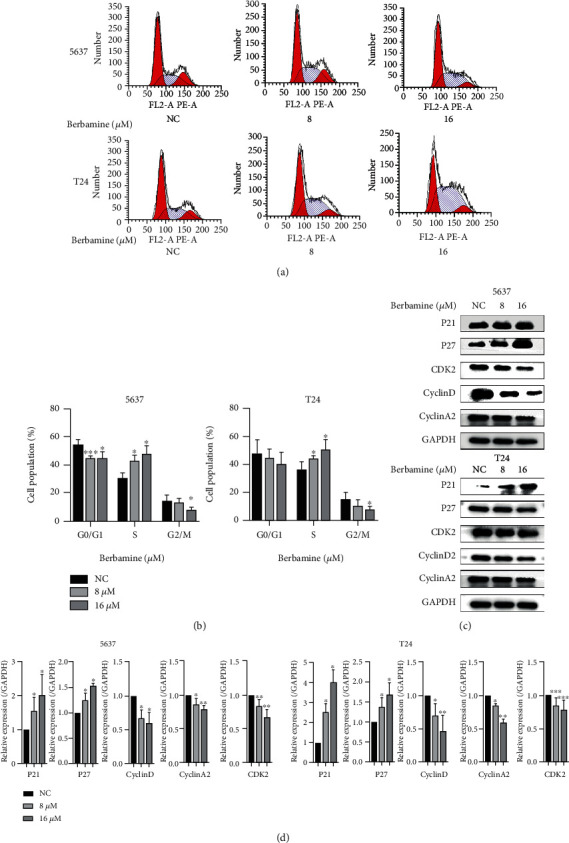
Berbamine induced S-phase arrest in bladder cancer cells. (a, b) Representative images and quantitative cell cycle distribution was detected by flow cytometry. (c, d) The protein levels of a cell cycle regulator involving P21, P27, CyclinD, CyclinA2, and CDK2 were examined by western blotting, and ImageJ analyzed relative expression levels. Values are represented (all dates are expressed) as the mean ± SD. The experiment was repeated at least three times. Statistical significance was determined using two-tailed Student's*t*-test or one-way ANOVA. ^∗^*p* < 0.5; ^∗∗^*p* < 0.01; ^∗∗∗^*p* < 0.001.

Cell cycle perturbation underlies aberrant cell proliferation, which characterizes a malignant phenotype [20]. Given that berbamine, a cycle-specific drug, could suppress tumor cell growth by disturbing cell cycle progression [8, 21], we measured the cycle ratio of 5637 and T24 cells with berbamine treatment by PI staining. As expected, berbamine increased the percentage of cells in S phase and exhibited a dose-dependent trend, but the proportion of cells in G0/G1 phase and G2/M phase did not change significantly (Figures 2(a) and 2(b)).

To clarify the molecular mechanism of how berbamine arrests the cell cycle, we assessed the levels of P21, P27, CyclinD, CyclinA2, and CDK2 proteins that are responsible for S-phase regulation [22]. As illustrated in Figures 2(c) and 2(d)), the expression of cyclin-dependent kinase inhibitors p21 and p27 was clearly upregulated upon berbamine treatment. In contrast, berbamine dramatically downregulated the expression of CyclinD, CyclinA2, and CDK2. In summary, berbamine induced S-phase arrest by targeting and altering the expression of checkpoint regulators, thus suppressing the growth of bladder cancer cells.

### 3.3. Berbamine Suppressed the Migration and Invasion Activities of Bladder Cancer Cells

**Figure 3:**
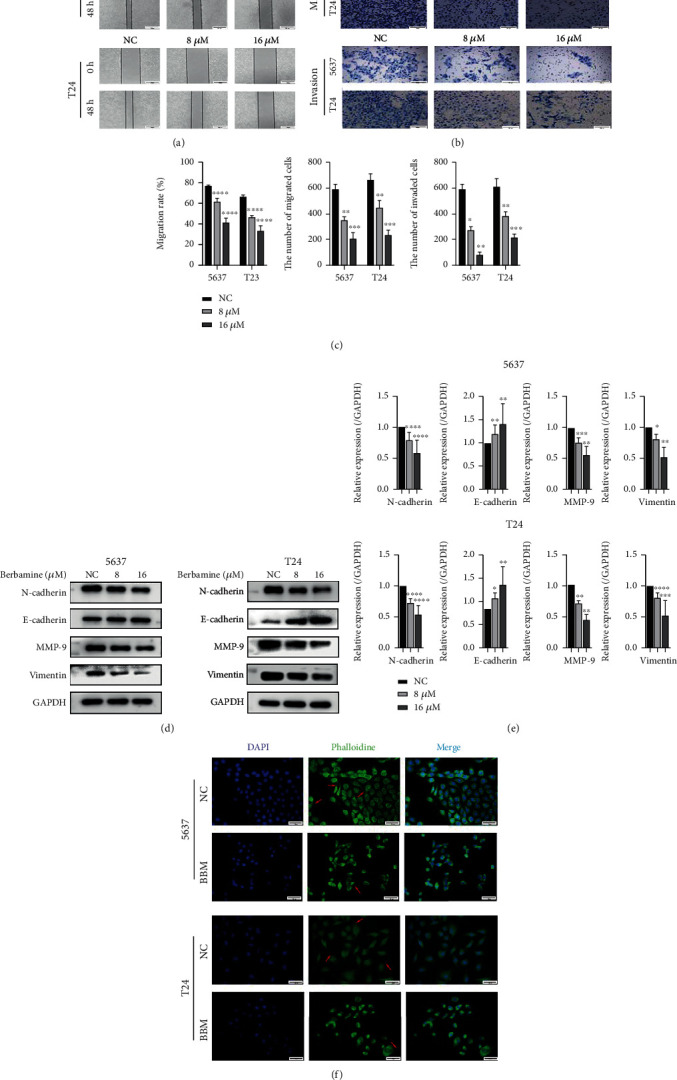
Berbamine inhibited migration and invasion of bladder cancer cell lines. 5637 and T24 cells were treated with the indicated concentrations of berbamine for 48 h. (a) Wound-healing assays were performed to evaluate the migration capacity. (b) Transwell assays with or without Matrigel were performed to evaluate the migration and invasion capacity. (c) The results of wound-healing assays and Transwell assays were analyzed using ImageJ. (d, e) The expression of EMT-related biomarkers was examined by western blotting. (f) Phalloidin dyeing of the F-actin cytoskeleton was performed to display morphological changes. The images were captured under inverted fluorescent microscopy. Values are represented (all dates are expressed) as the mean ± SD. The experiment was repeated at least three times. Statistical significance was determined using two-tailed Student's*t*-test or one-way ANOVA. ^∗^*p* < 0.5; ^∗∗^*p* < 0.01; ^∗∗∗^*p* < 0.001; ^∗∗∗∗^*p* < 0.0001.

Considering that the metastasis of cancer cells is a vital factor in tumor progression, we performed a wound healing assay and a Transwell assay to assess the influences of berbamine on the metastatic potency of bladder cancer cells. As shown in Figure 3(a), berbamine retarded wound closure in a dose-dependent manner, indicating that berbamine apparently curbed the migratory capacity of bladder cancer cells. Consistently, a similar result was obtained in the Transwell assay (Figure 3(b)). After treatment for 48 h, berbamine induced significant decreases in the numbers of migrated cells. Besides, the Transwell invasion assay revealed that the number of cells that invaded the lower chamber through extracellular matrix (ECM) gels was remarkably reduced following berbamine treatment, which suggested that berbamine restricted the invasive capacity of bladder cancer cells.

The above experiments confirmed the antimetastatic effects of berbamine on BCa cells (Figure 3(c)). Given that the EMT process has been verified to engage in the migration and invasion of cancer cells, we investigated the levels of select markers involved in EMT following berbamine treatment. Data in Figures 3(d) and 3(e) show that berbamine augmented E-cadherin expression and concomitantly decreased the levels of N-cadherin, vimentin, and MMP-9 in both cell lines. Therefore, the outcomes validated that berbamine attenuated cell metastasis by repressing the EMT process.

The remodeling of the actin cytoskeleton also plays a vital role in metastasis [23]. Filopodia are actin-based protrusions that mainly arise on the ventral surface of the cell membrane to assimilated signals like chemokines, nutrients, and chemoattractants [24, 25]. We next stained the cytoskeleton and pseudopodia with fluorescein-conjugated phalloidin. An interesting observation showed that 5637 and T24 cells without berbamine treatment maintained the spindle- and fibroblast-like appearance with lamellipodia at the cell perimeter. However, the treated cells exhibited a cobblestone-like morphology (Figure 3(f)), which was a feature of epithelial cells. Furthermore, there were few cellular protrusions and contact surfaces between cancer cells. In summary, it seemed clear that the inhibitory effects of berbamine on metastasis were also associated with cytoskeletal rearrangement.

### 3.4. Berbamine Inhibited the Biological Activities of Bladder Cancer Cells by Suppressing the NF-*κ*B Pathway

**Figure 4:**
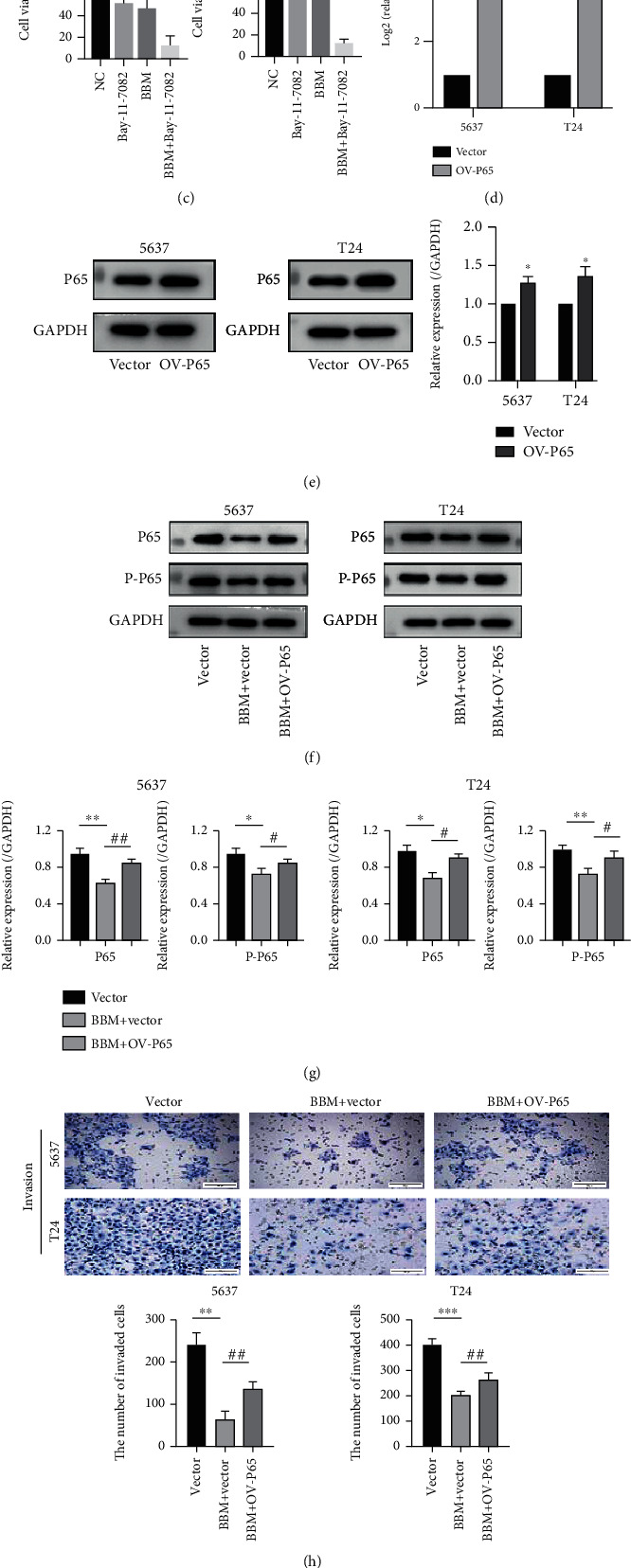
Berbamine inhibited the NF-*κ*B pathway in bladder cancer cell lines. (a, b) Western blotting experiments were performed to detect the expression levels of NF-*κ*B P65, P-P65, and P-I*κ*B*α* in both 5637 and T24 cells following berbamine treatment for 48 h. (c) A CCK-8 assay evaluated cell viability via a comparison between group exposures to 16 *μ*M berbamine and in the presence or absence of 10 *μ*M BAY-11-7082. (d, e) To detect the effectiveness of transfection, qRT-PCR and western blotting were performed to measure the expression of P65 at 48 h posttransfection. (f, g) Western blotting was performed to detect the expression of P65 and P-P65 in 5637 and T24 cells after berbamine treatment with or without pcDNA3.1-P65. (h) In rescue experiments, the invasive potency of 5637 and T24 cells was evaluated by Transwell assays with Matrigel following berbamine treatment with or without pcDNA3.1-P65. (i) EdU assays were performed to detect the proliferative ability of 5637 and T24 cells following berbamine treatment with or without pcDNA3.1-P65. Values are represented (all dates are expressed) as the mean ± SD. The experiment was repeated at least three times. Statistical significance was determined using two-tailed Student's*t*-test or one-way ANOVA. ^∗^*p* < 0.5; ^∗∗^*p* < 0.01; ^∗∗∗^*p* < 0.001; ^∗∗∗∗^*p* < 0.0001; ^#^*p* < 0.5; ^##^*p* < 0.01.

The exceptional NF-*κ*B pathway is known as a crucial participant in cell proliferation and EMT in bladder cancer, and blockade of the NF-*κ*B pathway could inhibit tumorigenesis and the progression of malignancies [26–28]. Previous studies have indeed identified berbamine as a novel inhibitor of the NF-*κ*B pathway with antitumor activity [29–31]. Thus, we hypothesized that the inhibition of NF-*κ*B activation is a potential mechanism by which berbamine interferes with the biological activities of bladder cancer. We measured the expression of the critical genes involved in the NF-*κ*B signaling pathway. As predicted, the levels of total P65, P-P65, and P-I*κ*B*α* were significantly decreased in the presence of berbamine (Figures 4(a) and 4(b)). To determine whether inactivation of the NF-*κ*B pathway caused by berbamine was responsible for the reduction in proliferation and metastasis, we initially chose the pathway-specific inhibitor BAY-11-7082 and then assessed cell viability by CCK-8 assay. Our results proved that BAY-11-7082 alone exhibited superior inhibitory activity and exacerbated cytotoxicity mediated by berbamine (Figure 4(c)). On the other hand, the pcDNA3.1-P65 plasmid was constructed and subsequently transfected into 5637 and T24 cells. As shown in Figures 4(d) and 4(g), the level of P65 dramatically increased, suggesting successful transfection. As expected, P65 overexpression partially abolished the inhibitory effect of berbamine on cell proliferation and metastasis in the rescue experiments (Figures 4(h) and 4(i)), which indicated that the antitumor action of berbamine against bladder cancer cells was mediated, at least in part, by inhibiting the activity of the NF-*κ*B signaling pathway.

### 3.5. Berbamine Triggered ROS Generation and Cell Apoptosis in Bladder Cancer

**Figure 5:**
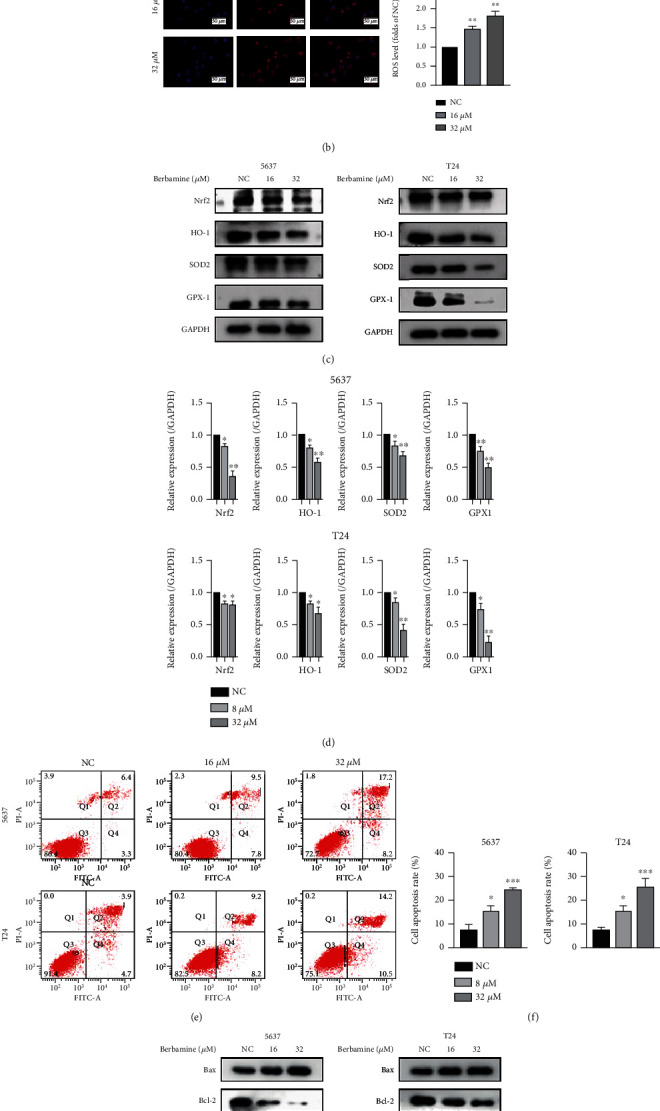
Berbamine-mediated ROS generation and apoptosis in bladder cancer cells. 5637 and T24 cells were incubated with 16 *μ*M and 32 *μ*M berbamine for 24 h. (a, b) MitoSOX fluorescence was performed to detect ROS generation. As a live-cell permeant fluorogenic dye, MitoSOX gets oxidized by mitochondrial superoxide to exhibit red fluorescence. Relative fluorescence intensity was analyzed by ImageJ. (c, d) The effects of berbamine on the levels of several antioxidant genes. (e, f) Flow cytometry was performed to determine the apoptotic percentage. (g, h) The effect of berbamine on the expression of Bcl-2 and Bax proteins. Values are represented (all dates are expressed) as the mean ± SD. The experiment was repeated at least three times. Statistical significance was determined using two-tailed Student's*t*-test or one-way ANOVA. ^∗^*p* < 0.5; ^∗∗^*p* < 0.01; ^∗∗∗^*p* < 0.001.

Mitochondria are vital sources of intracellular ROS involved in the regulation of diverse pathophysiologic processes [32]. In addition, fluctuations in ROS levels could regulate the proliferation and apoptosis of cancer cells in response to multiple stimuli [33]. Due to their short half-lives, we evaluated the changes in ROS levels of 5637 and T24 cells incubated with 16 *μ*M and 32 *μ*M berbamine for 24 h. MitoSOX images (Figures 5(a) and 5(b)) showed that red fluorescence intensity was remarkably elevated, indicating that berbamine directly accelerated the generation of mitochondrial superoxide. In most cells, the level of ROS strictly depends on the dynamic equilibrium between ROS generation and antioxidant systems. Next, we detected the expression levels of a few antioxidative genes of bladder cancer cells following berbamine treatment. The Nrf2, HO-1, SOD2, and GPX-1 genes were substantially downregulated (Figures 5(c) and 5(d)), which explained that the ROS accumulation mediated by berbamine is associated with the deficiency of antioxidant defense.

We further used the Annexin V-FITC/PI double-staining method with flow cytometry to elucidate the cytotoxic effect of berbamine on BCa cells in more detail. As shown in Figures 5(e) and 5(f), an obvious increase in the apoptosis rate was found upon treatment with a higher concentration of berbamine. To understand the molecular evidence of berbamine-induced apoptosis, we analyzed the expression levels of Bcl-2 family proteins that are master regulators of mitochondrial apoptosis. Western blotting results showed that berbamine dose-dependently increased the expression of the proapoptotic Bax protein while significantly inhibiting the level of the antiapoptotic Bcl-2 protein in both cell lines (Figures 5(g) and 5(h)). Overall, berbamine increased the Bax/Bcl-2 ratio, which is critical for the initiation of the intrinsic apoptosis pathway.

### 3.6. Berbamine Exerted Antitumor Activity against Bladder Cancer Cells by Modulating the ROS/NF-*κ*B Axis

**Figure 6:**
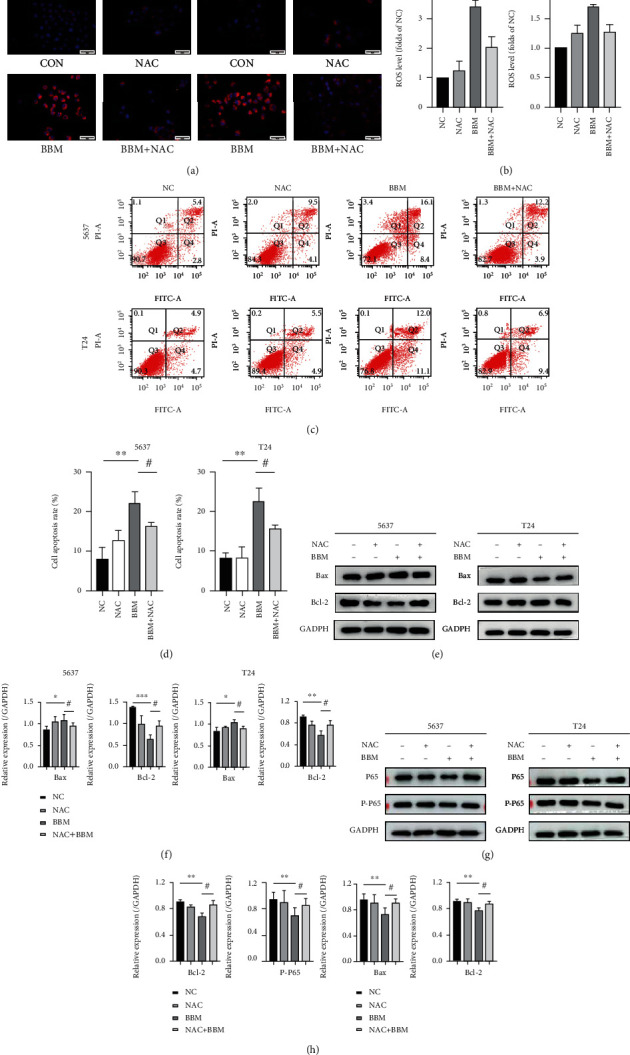
Berbamine exerted antitumor activity against bladder cancer cells by modulating the ROS/NF-*κ*B axis. 5637 and T24 cells were treated with 32 *μ*M berbamine in the presence or absence of 10 mM NAC. (a, b) Representative images of the ROS generation level were captured using a fluorescence microscope. (c, d) Flow cytometry was performed to measure cell apoptosis. (e, f) The levels of Bcl-2 and Bax proteins were measured by western blotting. (g, h) The levels of P65 and P-P65 proteins were measured by western blotting. Values are represented (all dates are expressed) as the mean ± SD. The experiment was repeated at least three times. Statistical significance was determined using two-tailed Student's*t*-test or one-way ANOVA. ^∗^*p* < 0.5; ^∗∗^*p* < 0.01; ^∗∗∗^*p* < 0.001; ^#^*p* < 0.5; ^##^*p* < 0.01.

It is well known that numerous anticancer drugs trigger cell apoptosis via a ROS-dependent pathway [34, 35]. Accordingly, we further explored whether cell apoptosis caused by berbamine is directly relevant to ROS accumulation. To confirm this hypothesis, we applied N-acetylcysteine (NAC), a ROS scavenger, to the berbamine-treated group in advance. As shown in Figures 6(a) and 6(b), pretreatment with NAC partially prevented berbamine-mediated mitochondrial ROS generation, followed by a decrease in bladder cancer cell apoptosis (Figures 6(c) and 6(d)). Consistent with the flow cytometry assay results, western blotting analysis showed that NAC partially reversed the effects of berbamine on the protein levels of Bcl-2 and Bax (Figures 6(e) and 6(f)). Numerous articles have demonstrated that ROS can modify cell-signaling proteins to mediate multiple pathways. We further explored the correlation between ROS and NF-*κ*B. We observed that the ROS inhibitor counteracted the NF-*κ*B pathway suppression mediated by berbamine to a certain degree (Figures 6(g) and 6(h)). Taken together, these observations indicated that the ROS/NF-*κ*B axis plays a vital role in the antitumor activity of berbamine against bladder cancer.

### 3.7. Antitumor Effect of Berbamine In Vivo

**Figure 7:**
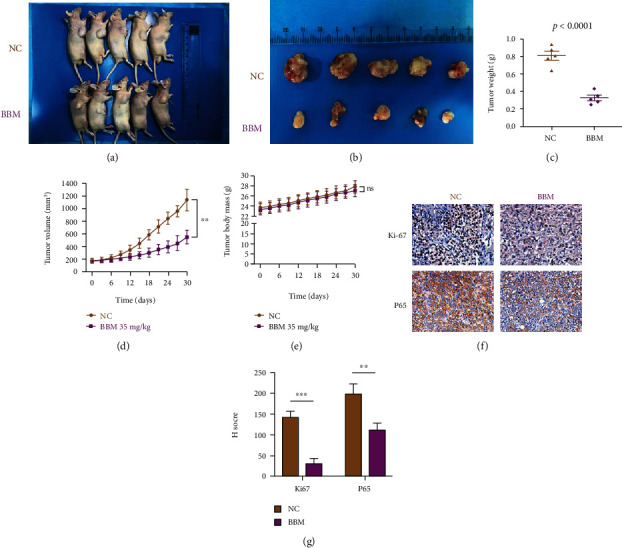
Berbamine inhibited the growth of the T24 xenograft tumor in vivo. Nude mice were treated with 35 mg/kg berbamine and the same DMSO via intraperitoneal injection every three days for 30 days. (a, b) Photographs of the tumors were taken at autopsy. (c) The weight of tumors in nude mice was measured. (d, e) The tumor volumes and body weight of nude mice were monitored every three days by the end of this experiment. (f) The levels of Ki-67 and P65 in tumor tissues were evaluated via IHC. (g) IHC staining score for Ki-67 and P65 protein. Values are represented (all dates are expressed) as the mean ± SD. The experiment was repeated at least three times. Statistical significance was determined using two-tailed Student's*t*-test or one-way ANOVA. ^∗∗^*p* < 0.01; ^∗∗∗^*p* < 0.001.

To further validate the antitumor effect of berbamine in vivo, we established a human-T24 subcutaneous xenograft in nude mice and recorded mouse weight and tumor weight throughout the experiment. As exhibited in Figures 7(a) and 7(b), berbamine exerted apparent cytotoxicity against cancer cells in vivo. The tumor volume and weight of the berbamine-treated group grew much more slowly than those of the control group (Figures 7(c) and 7(d)); however, there was no significant difference in the average weight of mice between the control group and the berbamine-treated group (Figure 7(e)), suggesting that berbamine possibly had no evident adverse effects in vivo.

IHC analysis showed that the positive rate of Ki-67 and P65 in the tumor tissue of the berbamine-treated group was significantly lower than that in the control group (Figures 7(f) and 7(g)), which is consistent with our in vitro results. Overall, our results suggested that berbamine reduces tumor growth and suppresses NF-*κ*B signaling in vivo.

## 4. Discussion

Bladder cancer is the most common malignant tumor of the urinary system, with high incidence and recurrence. Thus far, studies have extensively reported the pathogenesis and current therapeutic strategies of bladder cancer, consisting of surgical resection, immune checkpoint inhibition, and a combination of chemotherapy drugs. However, there are few studies on Chinese traditional medicine applications in clinical cancer therapy, which is possibly due to a lack of a comprehensive understanding of their mechanisms and safety. Berbamine is one of the active ingredients extracted from the herbal medicine *Berberis* and possesses multiple biological activities, including immunomodulatory, antihypertensive, and cardioprotective properties [36–38].

Natural compounds and their derivatives extracted from traditional Chinese herbs can be considered as ideal alternative anticancer agents owing to their lower cost, stronger effectiveness, and minimal side effects [11, 39]. Berbamine has exhibited favorable antitumor potential in previous studies, as it modulates various molecular targets and has low cytotoxicity in normal cells. For instance, berbamine suppressed cell growth and invasion ability while inducing G0/G1 cell cycle arrest and apoptosis by inhibiting Wnt/*β*-catenin signaling in ovarian cancer [9]. Additionally, berbamine exerted antitumor effects in vitro and in vivo through apoptosis induction partially relevant to the activation of the p53 gene in colorectal cancer [8]. Moreover, berbamine enhanced the efficacy of gefitinib in pancreatic cancer cells and radiosensitivity for head and neck squamous cell carcinoma by inhibiting the STAT3 pathway [40, 41]. Herein, we attempted to unambiguously investigate the effects and potential mechanisms of berbamine in bladder cancer in vitro and in vivo.

Cancer-cell-based experiments manifested that berbamine inhibited cell viability and impaired the colony formation ability of bladder cancer cells. Also, the EdU and Ki-67 immunofluorescence assay collectively revealed the antiproliferative effect of berbamine on bladder cancer.

Cell cycle deregulation leads to infinite cell proliferation, which is an elementary characteristic directly related to tumor progression. Thus, targeting the cell cycle pathway is emerging as a fundamental strategy to arrest neoplastic processes [33, 42]. The data of the present study demonstrated that berbamine induced significant S-phase arrest in bladder cancer cells. Regulations of the cell cycle are tightly dependent on the coordinated activity of protein kinase complexes that consist of cyclins, cyclin-dependent kinases (CDKs), and endogenous inhibitor proteins (CKIs). Progression through G1 phase is driven by activation of the CyclinA-CDK2 complex, and CyclinA is required for DNA replication throughout the S phase [43, 44]. To our knowledge, P21 and P27, inhibitors of CDKs, bind to these Cyclin-CDK complexes and induce their inactivation, thus halting cell cycle progression [45]. Concurrently, we observed that berbamine dramatically downregulated the expression of CyclinD, CyclinA2, and CDK2 and upregulated the levels of P21 and P27, which indicated that berbamine-induced S-phase arrest was mainly driven by enhanced initiation of S phase and concomitant suppression of S-phase progression.

Metastasis, the property that enables individual cancer cells to spread into local or distant tissues [46], remains a stumbling block limiting the effective therapy of bladder cancer and is also the leading cause of cancer mortality. EMT is a critical process responsible for the acquisition of malignant phenotypes in epithelial tumor cells [47]. In this process, cancer cells could lose cell adhesion attributes and acquire cytoskeletal activation [48]. Furthermore, EMT can endow tumor cells with stem cell characteristics, thus resulting in a poor prognosis for cancer patients [49]. Activation of NF-*κ*B is correlated with the induction of a well-defined set of transcription factors involved in EMT, such as Snail, Slug, Twist, and ZEB1/ZEB2 [50]. It was reported that NF-*κ*B P65, as a transcriptional activator, facilitated Snail transcription by directly binding to the promoter [51]. MMP-9 plays a crucial role in the invasive process of various solid tumors by degrading the extracellular matrix barrier [52]. The promoter of MMP-9 has been characterized as having a series of functional enhancer element-binding sites, such as NF-*κ*B and activator proteins (AP-1) [50]. Therefore, the inactivation of NF-*κ*B could decrease the basal transcriptional activity of the MMP-9 promoter, thus inhibiting the expression of the MMP-9 protein. Interestingly, we observed that berbamine could remarkably dampen the migration and invasion capacity of both cell lines. Western blotting revealed that berbamine increased E-cadherin expression while decreasing N-cadherin, vimentin, and MMP-9 expression. In our rescue experiment, P65 overexpression increased the number of invasive cells among those treated with berbamine. Thus, we postulated that berbamine might suppress the metastatic ability of bladder cancer cells through reversal of NF-*κ*B-mediated EMT. Moreover, berbamine impaired the cytoskeletal organization of 5637 and T24 cells. The cytoskeleton changed into an epithelial morphology upon exposure to berbamine, which could facilitate tight adhesion to avoid cell metastasis.

Apoptosis, also known as type I genetically programmed cell death, is a normal physiological process that accompanies morphological and biochemical changes involving DNA fragmentation, chromatin condensation, and membrane blebbing. To the best of our knowledge, apoptosis can be activated by either the extrinsic pathway initiated by the death receptor or intrinsic pathway through the mitochondria to prevent tumor formation. In our results presented here, berbamine elevates bladder cancer cell apoptosis in a dose-dependent manner. Previous studies confirmed that members of the Bcl-2 family, as key regulatory factors of the mitochondrial-mediated pathway, play an essential role in the antiapoptosis response [53–55]. Both Bax and Bcl-2 belong to the Bcl-2 family, and the ratio of Bax/Bcl-2 is relevant to the sensitivity or resistance of cancer cells to apoptotic stimuli and therapeutic drugs [56]. Western blotting revealed that berbamine reduced the level of the Bcl-2 protein but increased the level of the Bax protein. In a word, berbamine activates the mitochondrial-dependent apoptotic pathway by targeting the Bcl-2 family to exert a cytotoxic effect on bladder cancer cells.

Strict control of ROS levels is vital to regulate cell repair, survival, and differentiation [57]. Several lines of evidence highlight that once the redox status deviates to oxidation, the increased ROS function as redox messengers to accelerate the early events involving tumorigenesis and tumor progression. Mechanistically, as an upstream factor, ROS mediate DNA mutations and modulate various cellular signaling pathways, thus affecting several cancer hallmarks of metabolic reprogramming, angiogenesis, metastasis, and drug resistance development [58]. However, when the continued increase in ROS levels overwhelms intracellular antioxidant capacity, it can stimulate cell cycle arrest and cellular apoptosis [59]. Compared to normal cells, tumor cells are more sensitive to fluctuations in ROS levels, and excessive ROS induction is a common mechanism by which various antitumor agents scavenge cancer cells [60, 61]. ROS-mediated apoptosis is known to open the permeable transition pore of the mitochondrial membrane with the release of cytochrome c by regulating Bcl-2 family genes [62]. Representative MitoSOX images initially demonstrated that berbamine dose-dependently accumulated mitochondrial ROS in both cell lines. Not surprisingly, previous studies reported that berbamine, as a prooxidant, effectively induced intracellular ROS generation, thus enhancing the sensitivity of glioma cells to paclitaxel therapy [63]. It indicated that berbamine might modulate ROS levels to influence the biological behaviors of cancer cells. To further explore the mechanism underlying ROS generation, we measured the expression of a few crucial antioxidative genes. Western blotting results suggested that the levels of the Nrf2, HO-1, SOD2, and GPX-1 genes were downregulated following berbamine treatment, which implies that berbamine impaired the functions of the antioxidant system, followed by ROS production. Intriguingly, the apoptosis caused by berbamine was mitigated following the use of the antioxidant NAC, along with the reversal of Bcl-2 and Bax expression. As stated previously, excessive ROS generation upon the tolerable threshold plays a crucial role in the proapoptotic effect of berbamine on bladder cancer.

In response to multiple stimuli, aberrant activation of the NF-*κ*B signaling pathway participates in multiple malignant transformation processes by mediating the downstream oncogenic genes. However, earlier research confirmed that the NF-*κ*B pathway is constitutively activated in bladder cancer and is associated with muscle-invasive clinical features [64]. Herein, western blot analysis showed that berbamine negatively regulated the expression of P65, P-P65, and P-I*κ*B*α*, which indicated that berbamine blocked the NF-*κ*B signaling pathway. However, it remains unclear whether inactivation of the NF-*κ*B signaling pathway is sufficient to influence the progression of BCa cells. Suppression of NF-*κ*B activity by the specific inhibitor BAY-11-7082 dramatically inhibited cell viability in both cell lines assessed. Meanwhile, our rescue experiment revealed that cell proliferation was reversed following P65 overexpression. In addition, the activity of NF-*κ*B function can be regulated by ROS in different contexts [65]. NF-*κ*B activation is associated with ROS-mediated oxidation and activation of inhibitors of NF-*κ*B (I*κ*B) kinases, which negatively control the stability of I*κ*B. On the other hand, ROS inhibit NF-*κ*B transcriptional activity by interfering with NF-*κ*B DNA binding owing to the presence of oxidizable cysteines in the DNA-binding region [66]. In this paper, we identified a relationship in which ROS generated by berbamine acted as an upstream molecule to partially inhibit the NF-*κ*B pathway, which implies the indispensable role of the ROS/NF-*κ*B axis in berbamine-mediated antitumor activities against BCa cells.

Finally, a xenograft mouse model was established to further determine the inhibitory growth effects of berbamine in vivo. Consistent with the promising results in vitro, berbamine could reduce the tumor volume and weight. However, there was no significant difference in the average weight of mice between the control group and the berbamine-treated group, indicating that berbamine probably has no evident side effects in vivo. The results of human pharmacokinetic studies revealed that the half-life of berbamine was 39.25 h in the body, indicating that the compound's elimination was slow [67]. Thus, berbamine could be accumulated to an efficient concentration in vivo, although lower-dose drugs are administered. Further clinical studies are needed to explore a better dosage regimen. As indicated by IHC, the positive staining rate of Ki-67 and P65 frequently declined in the tumor tissue after berbamine treatment, which suggests that berbamine suppresses tumor growth and NF-*κ*B signaling in vivo.

## 5. Conclusion

**Figure 8:**
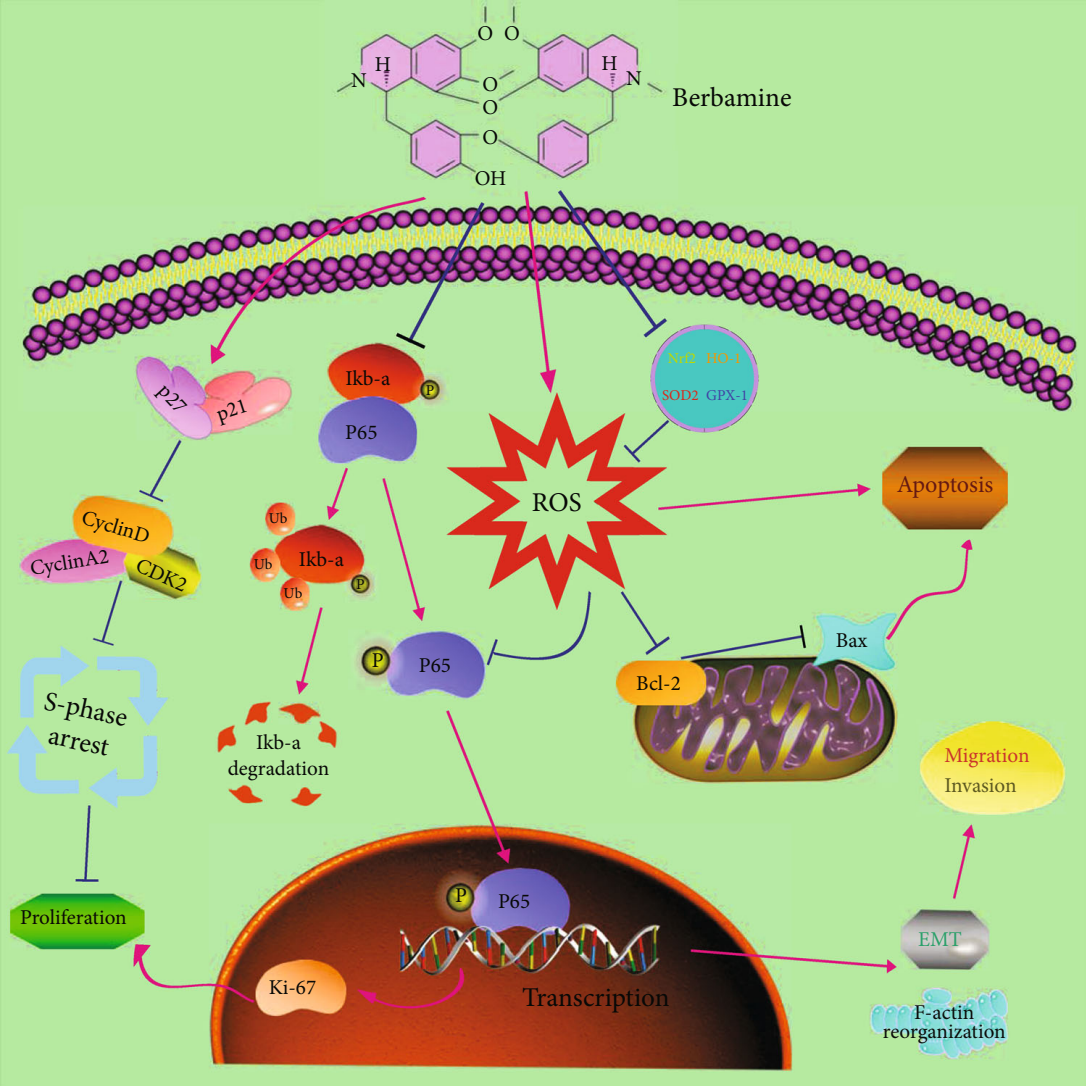
The potential mechanisms of berbamine occurring in bladder cancer cells.

We elucidated for the first time that berbamine could exert antitumor activities in bladder cancer by inhibiting cell proliferation and metastasis and inducing cell cycle arrest at S phase in vitro. Further analysis highlighted that berbamine suppresses the aberrantly active NF-*κ*B signaling pathway to interfere with the progression of bladder cancer. In addition, ROS accumulation induced by berbamine contributes to the intrinsic apoptosis of bladder cancer cells and inhibits the NF-*κ*B pathway to some extent (Figure 8). Finally, our in vivo experiments corroborate our in vitro findings. Based on the above results, it can be concluded that berbamine has potential clinical applications for patients with bladder cancer. Future studies are encouraged to ensure its drug safety and clarify its broader mechanisms.

## Data Availability

The data used to support the findings of this study are available from the corresponding authors upon request.
