# Erratum to “Berbamine Suppresses the Progression of Bladder Cancer by Modulating the ROS/NF-*κ*B Axis”

**DOI:** 10.1155/2021/9857803

**Published:** 2021-12-14

**Authors:** Chenglin Han, Zilong Wang, Shuxiao Chen, Lin Li, Yingkun Xu, Weiting Kang, Chunxiao Wei, Hongbin Ma, Muwen Wang, Xunbo Jin

**Affiliations:** ^1^Department of Urology, Shandong Provincial Hospital, Cheeloo College of Medicine, Shandong University, Jinan, Shandong 250021, China; ^2^Department of Vascular Surgery, Shandong Provincial Hospital, Cheeloo College of Medicine, Shandong University, Jinan, Shandong 250021, China; ^3^Department of Orthopedics, Shandong Provincial Hospital, Cheeloo College of Medicine, Shandong University, Jinan, Shandong 250021, China; ^4^Department of Urology, Shandong Provincial Hospital Affiliated to Shandong First Medical University, Jinan, Shandong 250021, China; ^5^Department of Hepatobiliary, The First Affiliated Hospital of Harbin Medical University, Harbin, Heilongjiang 150000, China

In the article titled “Berbamine Suppresses the Progression of Bladder Cancer by Modulating the ROS/NF-*κ*B Axis” [1], the incorrect figure files were used during the production process. The figures should be corrected as follows:

## Figures and Tables

**Figure 1:**
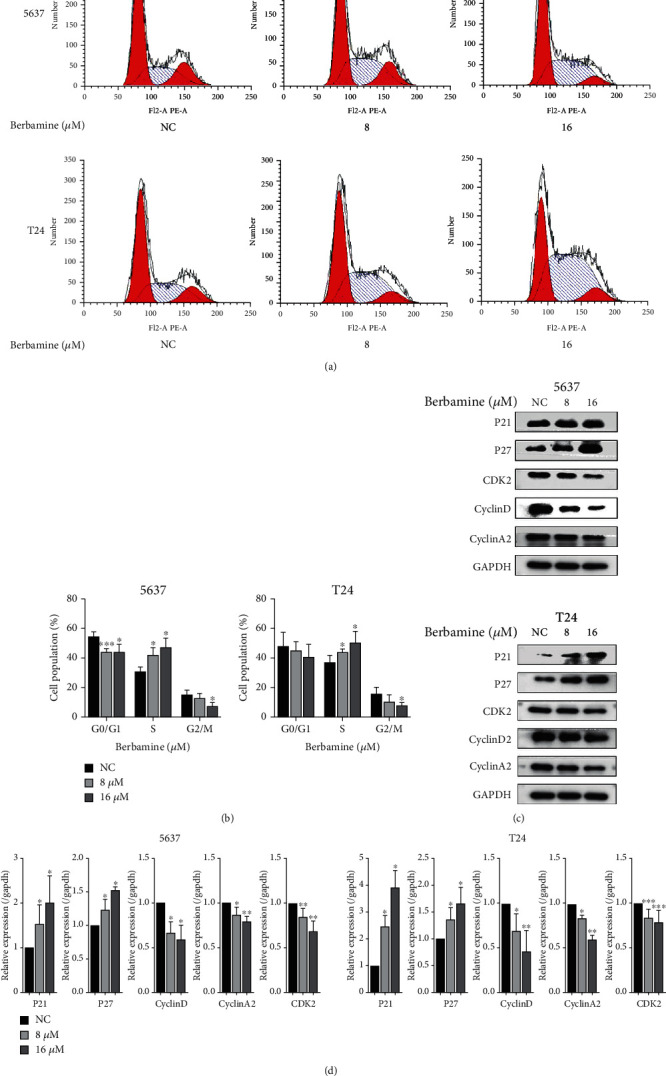
Berbamine induced S-phase arrest in bladder cancer cells. (a, b) Representative images and quantitative cell cycle distribution was detected by flow cytometry. (c, d) The protein levels of a cell cycle regulator involving P21, P27, CyclinD, CyclinA2, and CDK2 were examined by western blotting, and ImageJ analyzed relative expression levels. Values are represented (all dates are expressed) as the mean ± SD. The experiment was repeated at least three times. Statistical significance was determined using two-tailed Student's *t*-test or one-way ANOVA. ^∗^*p* < 0.5; ^∗∗^*p* < 0.01; ^∗∗∗^*p* < 0.001.

**Figure 2:**
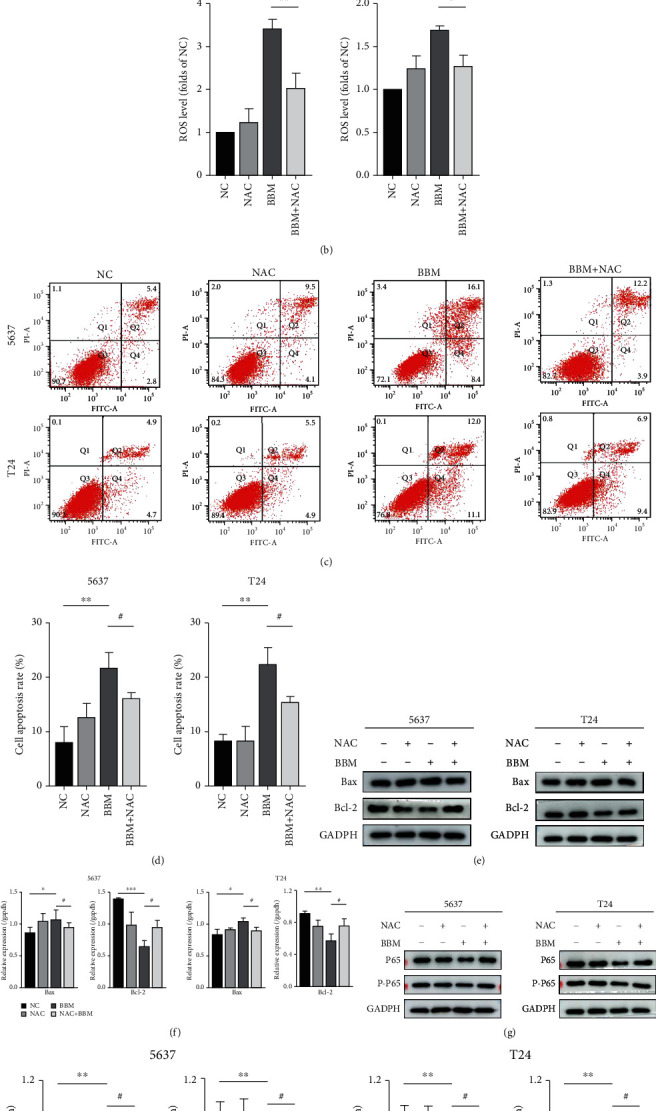
Berbamine exerted antitumor activity against bladder cancer cells by modulating the ROS/NF-*κ*B axis. 5637 and T24 cells were treated with 32 *μ*M berbamine in the presence or absence of 10 mM NAC. (a, b) Representative images of the ROS generation level were captured using a fluorescence microscope. (c, d) Flow cytometry was performed to measure cell apoptosis. (e, f) The levels of Bcl-2 and Bax proteins were measured by western blotting. (g, h) The levels of P65 and P-P65 proteins were measured by western blotting. Values are represented (all dates are expressed) as the mean ± SD. The experiment was repeated at least three times. Statistical significance was determined using two-tailed Student's *t*-test or one-way ANOVA. ^∗^*p* < 0.5; ^∗∗^*p* < 0.01; ^∗∗∗^*p* < 0.001; ^#^*p* < 0.5; ^##^*p* < 0.01.
